# Book Review

**Published:** 1910-03

**Authors:** 


**Medical Ethics.** By ROBERT SAUNDBY, M.D. Second Edition. Pp. 139. London: Charles Griffin & Co. 1907.—The recently-qualified medical man is liable to make grave ethical mistakes at the beginning of his career, either through ignorance or through having given insufficient consideration to apparently small matters during his early struggle to build up a practice. His friendly relations with his colleagues and his own peace of mind may be sadly disturbed through these mistakes, and it may take him many years to live down the results of these early slips. Professor Saundby gives annually a course of lectures on medical ethics in the Birmingham University, an example which might profitably be followed by all Universities. At the present time the public are more than inclined to scoff at medical ethics, which they term professional etiquette, and appear to be quite ignorant of the fact that they have more to gain than the medical profession has by the strict observance of professional etiquette. Senior medical students and all qualified medical men, but especially those recently qualified, should possess and read carefully this small book by Professor Saundby. The subject-matter of the book is arranged in alphabetical order, and there is a full index, so that there is no difficulty in looking up a required point. Obviously the range covered must be wide, and some subjects obtain too little attention: thus we should have liked to have seen more on the difficult position of the *locum tenens;* but for its size this book is admirable, and covers the great majority of difficulties that are likely to arise. Moreover, it is very interesting reading. We read it with some of that feeling of satisfaction with which we listen to a sermon directed against the sins of our friends, and sincerely hoping that they will take it to heart and profit by it.

**A Short Practice of Aural Surgery.** By J. ARNOLD JONES, M.B., B.Ch. Pp. viii, 264. London: John Lane. 1908. —The author has succeeded well in his attempt to give in this book a brief and concise account of the principles and practice of modern aural surgery. The text is not distinguished by any strikingly new departures either in arrangement, style or matter; but it is very readable. The paragraphs upon treatment are good and practical, especially those upon the after-treatment of operation cases, and they will be well appreciated by those readers who need guidance in such matters. A curious feature is an appendix giving German medical terms, used in relation to aural surgery, for the use of those who visit German clinics. The ordinary student or practitioner who masters the contents of this volume and takes a course of practical work in an English aural clinic will have little need to visit German clinics.

**Fourth Annual Report of the Henry Phipps Institute for the Study, Treatment and Prevention of Tuberculosis.** Edited by JOSEPH WALSH, A.M., M.D. Philadelphia. 1908.—The Annual Report affords ample testimony to the excellent and scientific work that is being carried out at the Institute, and the volume teems with valuable information on the many aspects of the problems yet to be solved in connection with tuberculosis. We are told that one of the functions of the Phipps Institute is to gather clinical and sociological data for scientific deductions, and the Institute has records, very carefully and accurately compiled, of nearly 5,000 cases, though still more interesting are the records of researches undertaken by members of the staff, for which they deserve to be congratulated.

**Dictionary of Ophthalmic Terms, with Supplement.** By EDWARD MAGENNIS, M.D., D.Ph. Bristol: John Wright & Sons, Ltd. (1909).—A booklet of sixty-seven pages, in which will be found some ophthalmic terms which are not in the ordinary medical dictionary. From it we learn that "light is one of the great fundamental powers of nature, by the action of which objects are made visible." We observe also that the term Lithiasis implies "callosity within the eyelid." The supplement contains many points worth remembering, and formulæ for the common diseases of the eye. The reader will find here a prescription for "black eye!"

**A System of Clinical Medicine.** By THOMAS DIXON SAVILL, M.D. Lond. Second Edition. Pp. xxvii, 963. London: Edward Arnold. 1909.—This new edition has been revised by the author, with the assistance of Dr. Fredk. S. Langmead and Dr. Agnes F. Savill. The press notices of the first edition welcomed the book so warmly that a second soon became necessary, and it has now been out of print for two years. It deals with the diagnosis, prognosis and treatment of disease for the purpose of utility to the student and the general practitioner, by approaching the subject from the point of view of symptoms and physical signs. This edition appears as one volume instead of two, but the amount of material remains approximately as before. Of the special features of the work we may mention the first part of each chapter dealing with symptoms and their causes. Great labour has been expended thereon, but it could not fail to be exceedingly profitable to the writer. Part B treats of the physical signs of disease in the special regions, and the various methods used to elicit them. Part C constitutes the major portion of each chapter, and contains a clinical classification of the various maladies affecting the particular region, followed by a series of sections dealing with the several diseases arranged according to their clinical relationships. The author remarks that " the advantages of passing in rapid review all the possible diseases which may give rise to a patient's leading symptoms are very obvious to those actively engaged in clinical work." The compilation of a work such as this must have been a gigantic task to the author, whose readers can now enjoy at comparative ease the fruit of his labour, and cannot fail to profit thereby.

**Gout.** By Prof. Dr. H. Strauss. Pp. 70. Bristol: John Wright & Sons, Limited. 1909.—This essay forms Part VIII of a series of clinical treatises on the pathology and therapy of disorders of metabolism and nutrition by Prof. Dr. Carl von Noorden, edited by Boardman Reed, M.D., of Philadelphia. Dr. Strauss has endeavoured to give, from the practitioner's standpoint, a concise picture of the modern conceptions of the nature and treatment of gout. It is by no means an easy task to enucleate the kernel from the shell in dealing with the voluminous literature of gout and uric acid, for uric acid appears in this monograph to be the essential factor of gout, and we are delighted to see the thread test of Sir Alfred Garrod again revived and occupying the diagnostic position which it held some forty years ago. The subject of uricacidæmia is briefly discussed, and its association with chronic interstitial nephritis is emphasised. " For the modern clinician, nephritis should be diagnosed before there is demonstrable albuminuria " (page 29).

**The Operative Treatment of Chronic Constipation.** By Arbuthnot Lane, M.S., F.R.C.S. Pp. 70. London: James Nisbet & Co. Limited. 1909.—In the latest edition of his book Mr. Lane adds very little that is new. He does not attempt seriously to deal with the objections which have been urged against his ideas of pathology, beyond a brief reference to a part of Mr. Hey Groves's recent work. That there is often found the co-existence of three classes of phenomena, viz. (1) chronic constipation, (2) abnormalities of the intestine both in position and peritoneal fixation, (3) various nervous and toxic phenomena, no one has ever denied. But which of these is the primary factor, and whether the others are caused by it, is open to much difference of opinion. Mr. Lane's pamphlet is scarcely more than a rather unconvincing formulation of an unproved theory, and after all these years of practical application and operations we have a right to expect the description of actual clinical cases and results. But this right is so far denied to us. In lists of cases published by Mr. Lane elsewhere we are struck by the facts (1) that a large proportion of his cases are those of chronic gastric ulcer and local peritonitis; (2) that many of these have given a very poor result after ileo-sigmoidostomy, and have recovered well after the stomach or appendix lesion have been directly treated; (3) there are no details given which enable any opinion to be formed as to the after-effect of ileo-sigmoidostomy or excision of the colon. This reticence over clinical details will operate even more strongly than the doubtfulness of his pathological theories in making the profession accept Mr. Lane's conclusions with great hesitation, if not actual scepticism.

**Manual of Diseases of the Ear.** By THOMAS BARR, M.D., and J. STODDART BARR, M.B., C.M. Fourth edition. Pp. xxvii, 477. Glasgow: James Maclehose & Sons. 1909.—We welcome this new edition of one of the most deservedly popular treatises on diseases of the ear in the English language, for the authors have succeeded admirably in their efforts to bring the present work up to date, and to include in it the advances which have been made since the third edition was published. The two chapters on the nose and throat, and especially that on the nose in its relation to affections of the ear, are of much value, and the subject is treated with sufficient fulness and detail. From cover to cover the volume is a masterly and well-balanced treatise, which does credit to the reputation of the authors, and, we may add, to the printer and publisher.

**Golden Rules of Anæsthesia.** By R. PROBYN-WILLIAMS, M.D. Third Edition. Bristol: J. Wright & Sons, Ltd.—The revision of this edition has not led to any very noticeable changes in its contents. We are sorry that room was not found for a short description of the open ether method. Certain drawbacks are inseparable from such concentrated forms of writing as is represented by this little book, *e.g.* on page 48 in discussing the care of the patient after ether anæsthesia the author gives the impression that the one remedy by which to treat shock is the administration of strychnine—something should surely have been said of saline, etc. On page 49 we notice a repetition of a mistake which occurred in the last issue. Ethyl-chloride before ether is recommended in doses of "three or four minims"—rather a difficult quantity to mete out.

**The unknown Life and Works of Dr. Francis Joseph Gall.** By BERNARD HOLLANDER, M.D. Pp. 31. Published for the Gall Society. London: Siegle, Hill & Co. 1909.—This pamphlet is a reprint of an address delivered before the Gall Society at its inauguration in May, 1909, and is a eulogy of "the discoverer of the anatomy and physiology of the brain." This description of Gall presumes, we venture to think, too far upon our suggested ignorance of his writings. As Dr. Hollander presents the case, it appears that Gall did in fact anticipate many of the modern discoveries, but in his own writings the proof is not by any means so clear that Gall formed any conception of cerebral localisation as now accepted. Nor did he stand alone in looking for a physiological basis for psychic activities even in his own day. There is more danger now of reviving his attractive errors than of reclaiming from his almost forgotten writings the hidden rays of illuminating truth. His fantastic materialisation of the abstract virtues and vices will meet with more ready acceptance at the hands of the credulous than will his occasionally accurate observations at the hands of the scientific. Moreover, there is some ground for thinking that the interest his writings arouse may be attributable to his tendency to deal with the psychopathia sexualis, which certain morbid minds find diverting reading. To our thinking his greatest claim to be remembered lies in the appreciation he won from the Combes, George and Andrew, who diligently adopted what was best in his teachings and had little interest in their charlatanry. Gall was not boycotted on this account, but for fear lest his impious tenets should oust " original sin " from the high place assigned to it by theologians, and bring it under the dispassionate analysis of scientists. Even Prichard was at fault in his forecast of the validity of Gall's view that a moral deterioration might be the result of a cranial injury, for he criticises the account of such a case, saying, " If stories of this kind gain credit, the College of Surgeons may expect one day to march in triumph and take possession of the vacant seats of the criminal judges; and we shall proceed forthwith to apply the trepan where now the halter and gibbet are thought most applicable." Andrew Combe, " preferring," according to Dr. John Brown, " observation to speculation," trenchantly retorts, " We confess that there are many instances in which we should not regret to see a well-informed and philosophical physician or physiologist taking his seat at the Bench along with the judges and benefiting them by his advice. The unfortunate, but innocent, insane would then have a still better chance of escaping the cruel fate which even in the present enlightened age not infrequently overtakes them; and our best feelings would be still more rarely outraged by seeing the victim of disease loaded with the infamy of guilt." To his contemporaries Gall's unpardonable offence lay in anticipating Haeckel's dictum " that every psychic action requires the complete and normal condition of the correlative brain structures for its full and normal exercise." To us he appears rather as an example of that type of frail humanity which condones its own incorrigible moral delinquencies by inveighing against the blind superstitions of the old religion. He lived an unlovely life, which the decadence of contemporary modernity may attempt to idealise in vain.

**Studies in Tuberculosis.** By HENRY CLARKE, M.A., M.D. Pp. 59. Liverpool: The University Press. 1909.—Dr. Clarke gives his experience of tuberculosis in three sections, according to his point of view as a physician, a city councillor, and a pathologist. The book is an amplification of a thesis for the Cambridge M.D. The most interesting portion of the book is where the author gives his experience in tuberculin treatment. He finds that the continental method, transgressing Wright's rules with regard to inoculation during negative phases, does not cause the ill results the opsonists threaten us with. The patient's temperature is the guide in the continental method, and is a sufficient guide with the dosage more generally used in this country. As a councillor he advocates segregation of the tuberculous and the destruction of infected cows. He rightly points out that much may be done with existing buildings in the formation of sanatoria, and additional buildings put up at a cost of £100, or at most £200, per bed, with little loss in efficiency compared with those costing anything from £500 to £1,000 per bed. The book will serve as a readable and simple introduction to the subjects discussed. The author pays a well-deserved tribute to Sir William P. Hartley, who has done so much to help Liverpool to deal with consumption.

**Open Air at Home : Practical Experience of the Continuation of Sanatorium Treatment.** By STANLEY H. BATES. Bristol : John Wright & Sons Ltd. 1910.—This booklet of 62 pages, with a prefatory note by Sir James Crichton-Browne, M.D., F.R.S., is intended for patients who have passed through the sanatorium, to whom it should prove exceedingly useful. We hold that no patient can carry out sanatorium life except in the sanatorium, but post-sanatorium treatment is most useful and necessary. The book describes the evolution of the garden shelter, and its uses. It cannot fail to be a good guide, not only to the patient on leaving the sanatorium, but to the patient's family and friends, to whom its injunctions should be a useful protective.

**Hypnotism and Treatment by Suggestion.** By J. MILNE BRAMWELL, M.B., C.M. Pp. xii, 216. London : Cassell & Co. 1909.—The numerous and conflicting theoretical considerations on hypnotism are dealt with in detail in the author's larger volume ; the present work is intended to give such practical information as may be valuable to those who wish to employ "suggestion" in their practice. The author is an enthusiast in his work, and be holds that suggestion ought to be a subject of keen interest not only to the physiologist and the psychologist, but also to the medical practitioner. Braid employed the term hypnotism because he believed the condition to be one of artificial sleep, but the author has long recognised that the condition differs widely from natural sleep, and that every stage of the so-called hypnotic condition is a conscious one, and the patient is often obviously wide awake. An enormous amount of clinical detail will be found in the book, and is sufficiently convincing as to lead any unbiased observer to the conclusion that "medical men can neither afford to ignore a legitimate and valuable form of treatment, nor to allow it to fall into the hands of unscrupulous and dangerous quacks."

**Experimental Psychology and Hypnotism.** By GEORGE H. SAVAGE, M.D. Pp. 44. London: Oxford University Press. 1909.—This was the principal topic of Dr. Savage's Harveian Oration, delivered before the Royal College of Physicians of London on October 18th, 1909. The author submits "that the investigation of hypnotism is a thing that should not be ignored in this country. When other nations are carefully investigating the physiology and the therapeutic value of this potent influence, it is certainly rather a pity that England should be in the background." He considers that whilst the supposed dangers of experimental hypnotic methods have been exaggerated, experience has shown him that hypnotism can only have a limited use, and that its use is not among the actively insane; but nevertheless, thoughtful men all over the world are recognising that it is a force to be considered. The author has obtained some encouraging results from the method of post-hypnotic suggestion.

**Rotter's Typische Operationen.** 8 Auflage. Von Dr. ALFRED SCHÖNWERTH. Pp. xii, 371. München: J. F. Lehmann. 1909.—The fact that this little book has reached its eighth edition probably indicates that it has attained some popularity among German medical students. It is somewhat a relief to find that our strenuous Teutonic cousins do allow themselves sometimes to write and to read a small summarised account of a scientific subject. Operative surgery is treated in a manner suitable for students and nurses, and great judgment has been exercised in selecting the most important principles, and in excluding that bewildering multiplication of methods which is so confusing to a learner. In very few respects does this German account differ from similar English books, but we note that Pirogoff's amputation of the foot is regarded as the typical operation, and Syme's as the exceptional. Nephropexy is dismissed in a few lines with the statement that this operation is now seldom performed! The illustrations are comparatively few in number, but are of large size and very clear in detail. A very useful set of pictures, illustrative of instruments in common use, ends the volume. The highest praise that can be given to the book is the statement that all the important procedures of operative surgery are clearly described, together with their chief indications, within the small compass of 362 pages. The book may be highly commended to the English reader who wishes to learn German for medical purposes. The sentences are short and much less involved than is the case with most German treatises.

**Soured Milk and Pure Cultures of Lactic Acid Bacilli in the Treatment of Disease.** By GEORGE HERSCHELL, M.D. Second Edition. Pp. vii, 72. London: Henry J. Glaisher. 1909.—The soured milk method has become so popularised that an *exposé* of its uses and limitations in disease is most desirable. The first edition having been rapidly exhausted a second revised and enlarged edition has become necessary. Of the preparations of lactic acid bacilli which now flood the market those at the disposal of the physician are practically three: Soured milk, liquid cultures of the Bulgarian bacillus, and dried cultures in the form of powder or tablets. The author points out that it is always best to make use of a pure laboratory culture, and that it takes about a week for the lactic germs to gain a footing, so that time must be given to allow the bacillus to do its work in the intestine.

**Some Common Remedies, and their Use in Practice.** By EUSTACE SMITH, M.D. Pp. vii, 112. London: H. K. Lewis. 1910.—This book consists of reprints of papers contributed to *The British Medical Journal* during the years 1908 and 1909, and is intended to show that the modern methods of treatment, founded on our knowledge of infection and the influence of micro-organisms, should not be allowed to supplant the older methods unduly. "Drugs have still their uses, and have not yet been superseded by antitoxic serums and vaccines." That is the text of the book, and the younger generation of practitioners will find that the making of a diagnosis is only the first step in a treatment, in which ignorance of general therapeutics is likely to be a source of continual embarrassment.

**House-drainage, Sewerage, and Sewage Disposal in Relation to Health.** By LOUIS C. PARKES, M.D., D.P.H. Pp. viii, 142. London: H. K. Lewis. 1909.—These lectures, delivered at the University of London under the Trust created by the will of the late Sir Edwin Chadwick, K.C.B., are perhaps better described by the official title: "The Medical Aspects of Recent Advances in Hygiene as connected with Sewering." They are well considered and interesting, and are fully up to date.

**Health, a Royal Road to it. A book for you. Read it: Try it.** By J. P. SANDLANDS, M.A., T.C.D. London: The Walter Scott Publishing Company Ltd. 1909.—Vicarious suffering is a great moral law. There must always be someone who is willing to suffer for others, or who is made to suffer for them. This is illustrated in the reviewing of this book. To save anyone else the pain of reading it, I have undertaken to review it, and I think it ought to atone for nearly all my sins. I have actually read it through, and I really think no one else need do so unless he or she is anxious to see what ineffable nonsense can be written by one whose "faith in himself" (as the *Lancet* justly remarks) "is colossal, and whose knowledge of medicine is *nil*."

